# Milk and Milk-Contracts
*Previous articles appeared on May 3 and 10.


**Published:** 1913-05-17

**Authors:** Everitt E. Norton

**Affiliations:** Medical Superintendent of Isleworth Infirmary.


### MILK AND MILK-CONTRACTS.*

By EVERITT E. NORTON, M.D., D.P.H., etc., Medical Superintendent of Isleworth Infirmary.

### III.—The Treatment of Institution Milk.

The results of inquiry clearly show that there is a general absence of faith in the purity of the milk supplied to London institutions and that, with much justification, it is regarded and treated as a damaged product. There have arisen, in consequence, customs of attempting to ameliorate after delivery conditions which have come to be regarded as unavoidable.

This distrust and suspicion are especially evidenced by the prevalent practice of making special arrangements for the supply of such milk as is required for infant-feeding. Apart from such special provision for infants I find that the milk when received is almost universally "treated"; in fact, as I have said, attempts are made to combat its suspected defects and dangers.

Straining is widely adopted, and is carried out by means of butter-muslin, wire-gauze, "filtrix cloth," or the "Ulax" filter. "Cleansing" by means of centrifugal apparatus, although it forms a part of certain processes which appear to be becoming increasingly common "in the trade," has not, so far, been practised in institutions. The milk is then commonly "pasteurised." Widely different methods are accepted as constituting "pasteurisation," that term, and "sterilisation" being very loosely used. In the apparatus employed, there are great variations in the degree of temperature attained, and the length of time of exposure to heat, and considerable vagueness and laxity are shown. In some cases the purpose in view—namely, that of delaying the souring of the milk, is naively made plain by the fact that the heating is only resorted to "during the summer months," "in hot weather," or even "in summer for delaying turning," or "one churn is scalded during summer months in case of emergency."

The apparatus used is frequently a steam-jacketed copper, a potato (or other) steamer, or (less frequently) one of the special apparatus of more complex and costly design made for the purpose. Certain of these latter are designed for rapid heating of the milk by a steam-heated coil or by plunging a steam-heated cylinder into the milk contained in a churn. In some institutions the heating process is carried out, by domestic methods, in the ward kitchens.

It appears that pasteurisation is generally taken to mean heating to any temperature between 160° F. and boiling-point; a temperature of 180° F. is frequently used, and a temperature of either 170° or 180° F. is defined in a majority of instances as that of pasteurisation. In a few cases the milk is boiled, is heated "to boiling-point," or the temperature to be reached is vaguely stated as "short of complete sterilisation."

The length of time during which the temperature is to be maintained is but seldom specified. Not uncommonly the milk is simply drawn off and is served to the wards as soon as the chosen temperature is reached. Very often the heating is so slow a process that there must be exposure to a temperature nearly approaching the maximum for some considerable length of time; in a few institutions the maximum temperature is maintained for a definite period of time, usually for five, rarely for as long as twenty, minutes.

Any routine process of subsequent cooling is uncommon. There is no doubt that a considerable quantity of the milk dealt with is needed for immediate distribution and consumption, and is consequently found to reach the wards at a temperature at which it can be conveniently consumed without further warming. Such of the supply as is not so needed is allowed to cool to the temperature of the air in covered vessels or is placed in ice-chests. Definite cooling is practised in some instances, when the apparatus in use permits, to a temperature of 80° or 100° F., this being sometimes effected very rapidly and sometimes occupying as long a time as an hour. Very rarely, the milk is further cooled by the use of brine to 40° F., being in such cases presumably not needed for immediate use.

Apart from any question as to the principles of pasteurisation or sterilisation the processes in use present in themselves certain points deserving of consideration. The methods are only too often ill-defined and therefore likely to be carried out in a haphazard way. The heating is either done under conditions in which the temperature attained cannot be observed, or the milk is exposed during heating to contamination, the exposure being the longer on account of the inordinate length of time required for heating in bulk. Some of the modern apparatus for pasteurisation, though in many ways excellent in construction, have two defects:—

(i) The heating surface may be raised to a temperature unduly high. The result is that, as convection in milk when heated is much less active than in water, the layers of milk in immediate contact with the heating surface are altered by scorching or burning. This can be, and often is, prevented by the provision of means for constantly stirring or maintaining a flow of the milk.

(ii) The milk may be heated and cooled again, unless the outfit contain special arrangements for maintaining the temperature for a given time, with such rapidity that, although it may show at one part of the apparatus a certain degree of heat, the actual exposure to that degree is momentary. The process then becomes one of "scalding" only, and uncertainty arises as to what effect, if any, in inhibiting the growth of, or destroying, organisms is attained. Dr. Eastwood calls attention to a similar variation and uncertainty in the methods employed by some milk-dealers in America, and quotes the milk-inspector of Boston as saying: "Commercial pasteurisation means the heating of

* Previous articles appeared on May 3 and 10.

milk to varying temperatures, usually from 155° to 170° F., for a short time, usually a matter of seconds, and then suddenly cooling . . . oftentimes this pasteurisation means that filthy milk which would under ordinary circumstances be unsaleable, because of souring, is given a new lease of life and becomes a marketable commodity."

It is hardly necessary to point out that steam should never, under any circumstances, be allowed to find its way into milk, or be passed into it for the purpose of heating.

The question of the means adopted in institutions for the storage of milk not needed for immediate use, whilst very important, is one which does not directly affect that of contract supplies. Whilst, however, deliveries under contract take place twice only in twenty-four hours it is obvious that milk must be stored and kept available for use from the time of one delivery until that of the next, which fact renders considerations of the age and condition of milk upon delivery of the greater importance.

